# Editorial: Rare and misdiagnosed neurosurgical conditions

**DOI:** 10.3389/fsurg.2023.1205968

**Published:** 2023-05-23

**Authors:** Hongyi Liu, Jian Wu

**Affiliations:** Beijing Institute of Ophthalmology, Beijing Tongren Eye Center, Beijing Tongren Hospital, Capital Medical University; Beijing Ophthalmology & Visual Sciences Key Laboratory, Beijing, China

**Keywords:** prognosis, adjuvant treatments, rare and uncommon neurological disorders, commonly misdiagnosed neurosurgical disorders, case

**Editorial on the Research Topic** Rare and misdiagnosed neurosurgical conditions

Be traced back to the ancient Incas and Hippocrates and through the renaissance and World Wars, the history of neurosurgery has been long (1). It was torturous at the same time since the outcome of surgery is worse than disease progression in the early stages of neurosurgery (2). However, the field has made significant strides through the development of clinical neurophysiology, advanced neuroimaging techniques (including Computed tomography, Magnetic resonance imaging, and Photon emission tomography), intraoperative magnification, and the establishment of neuro anesthesia as an independent specialty. These four factors have been key contributors to the remarkable progress in the field of neurosurgery (3). As the success rates of neurosurgical procedures continue to improve, it becomes increasingly important to prioritize the recognition and accurate diagnosis of rare and commonly misdiagnosed neurosurgical disorders, even if their incidence is lower than that of more prevalent conditions such as, glioma, and traumatic brain injury (4, 5). Moreover, the symptoms of many rare and misdiagnosed neurosurgical conditions often overlap with those of more common conditions, and the optimal therapeutic approach and perioperative management strategies for these disorders remain unclear. Accurately identifying and diagnosing these conditions can facilitate early intervention, which is crucial in preventing long-term damage and optimizing clinical outcomes.

As such, the research topic focus on the diagnosis, classification, surgical, adjuvant treatments, as well as prognosis of rare and misdiagnosed neurosurgical conditions. Based on this, the topic counts with total eight articles, comprising of six case reports and two original research.

As delineated by the research topic, this study has collated reports of severe medical conditions that were previously unreported. Zexin Cao et al. reported the first case of an aggressive angiomyxoma(AAM) in the skull of 2-year-old girl, typically manifests in the genital region of women of reproductive age. Ying Chingli et al. presented the inaugural case of intracranial cryptococcoma with middle cerebral artery (MCA) infarction in an immunocompetent patient. Jinchao Wang et al. reported the initial occurrence of a malignant triton tumor in the sellar region that was complicated by a concurrent relapsed pituitary tumor. Faisal Almatrafi et al. published a case report on an intradural extramedullary meningioma of the spinal cord that exhibited a rare extradural foraminal extension. Additionally, during glioma resection in a patient positive for syphilis, a cerebral herniation resulting from a gelatinous substance rather than a hematoma was observed, which had not been reported in prior surgical procedures.

For a more comprehensive understanding of diseases, scholarly articles on the topic have made significant contributions. Shiliang Cao et al. conducted a literature review and experience from a single center of surgical treatment of spinal tenosynovial giant cell tumor (TGCT). The study findings demonstrated that surgery is an effective treatment option for spinal TGCT and they explored the suitable section which is meaningful for TGCT treatment. Long Chen et al. present a study on the prognostic factors, clinical characteristics, and management of supratentorial hemangioblastoma based on relevant cases.

In clinical practice, due to the inherent limitations and the susceptibility of human disease cognition to habitual thinking biases, the reported case not only presents an infrequent occurrence but also offers potential avenues for diagnostic exploration by clinicians, and serves as a valuable reference for atypical cases. For example, AAW provides a new insight and possibility for neurosurgeons about the craniocerebral space–occupying lesions of unknown nature. the possibility of Cryptococcus infection ought to be taken into account in patients presenting with brain mass lesions alongside anomalous laboratory findings. Additionally, Hua Zheng et al. have reported a case of hyperkalemic cardiac arrest induced by mannitol during craniotomy. This case highlights the need for neurosurgeons to exercise caution when administering mannitol, which is the most frequently employed hypertonic agent in neurosurgical procedures, owing to its potential for causing hyperkalemia.

In conclusion, the papers incorporated in this topic have yielded certain advancements in the field of uncommon neurological disorders. They furnish reference for clinicians to undertake differential diagnoses and establish diagnoses, while concurrently broadening our comprehension of the ailment due to the emergence of these atypical cases.
